# Characterization of Microstructure, Precipitations and Microsegregation in Laser Additive Manufactured Nickel-Based Single-Crystal Superalloy

**DOI:** 10.3390/ma13102300

**Published:** 2020-05-16

**Authors:** Zhaoyang Liu, Jiayang Shu

**Affiliations:** 1 College of Innovation and Entrepreneurship, Southern University of Science and Technology, Shenzhen 518055, China; 2 Shenzhen Key Laboratory for Additive Manufacturing of High-Performance Materials, Department of Mechanical and Energy Engineering, Southern University of Science and Technology, Shenzhen 518055, China; jyang_shu@163.com

**Keywords:** single-crystal superalloy, microstructure, precipitations, microsegregation, laser additive manufacturing

## Abstract

In this study, the microstructure, precipitations, and microsegregation in the laser additive manufactured thin-wall structure of a single-crystal superalloy are synthetically characterized. The influence of a subsequent heat treatment on the microstructure and precipitations is discussed. The results show that under the given processing conditions, the single-crystal microstructure is regenerated perfectly with small misorientation angles in the thin-wall structure. The crystal morphology shows obvious diversity and instability with the incremental height of thin-wall structure. With the increase of manufacturing height, both the primary and secondary dendritic arm spacings of epitaxial columnar dendrites first increase rapidly and then reach a dynamic balanced state. The distribution of precipitations and pores keeps symbiosis in the interdendritic region and shows periodic band characteristic with high density in the band region and low density in the inner region of plate layers. The microsegregation of element atoms in the microstructure shows a three-dimensional network distribution. The concentration of element atoms keeps good consistency with high value in the three-dimensional network and nearly standard value in the outside region. The subsequent heat treatment process contributes to the occupation of as-processed pores by the expanded mature precipitations with good blocky shape. Further optimization of the heat treatment process for improving the lattice coherency of precipitated γ’ phase and γ matrix in the laser additive manufactured single-crystal superalloy is needed and valuable.

## 1. Introduction

To meet the rapidly increasing demand of raising the fuel efficiency of gas turbine engines, the high-temperature turbine blades with nickel-based single-crystal (SX) superalloy have been widely used to elevate the combustion chamber working temperature [1,2]. The monocrystalline nature of SX turbine blades supports superior high-temperature mechanical properties and simultaneously causes high crack susceptibility [3,4]. During the long service in the combustor, the lifespan of SX turbine blades is restricted by various unavoidable defects [5,6]. A common one is the tip material loss resulting from the abrasion between blades and engine shroud. Once the tip material loss reaches a certain amount, the blades need to be replaced. Within each aero-engine containing many high value SX turbine blades, the replacement of these disabled SX parts brings high operation cost and long maintenance time. Techniques for restoring the worn SX turbine blades back to parent metallurgy and microstructure at the damaged location and allowing these refurbished turbine blades to be reused rather than scrapped, are of great economic interest.

In recent decades, the laser additive manufacturing (LAM) process, also called Direct Energy Deposition [7], an layer-by-layer incremental forming technique capable of adding materials (powders/wires) accurately and rapidly with tailored microstructure, showed the tempting potential to refurbish worn tip of SX turbine blades in a near-net shape [8,9,10]. During the LAM process, a high-energy focused laser beam irradiates the base surface and creates a fast-moving molten pool continuously melting the base material and capturing the added materials. At the curved solidification interface (trailing edge of the molten pool), fine columnar crystals grow epitaxially with crystallographic orientation identical to the SX base and are interrupted by the ubiquitous formation of stray grains (SGs) [11,12,13,14]. A large number of SGs (mainly equiaxed grains), once they are not completely remelted by new beads and remain in the deposits, not only destroy the epitaxial-growth continuity of columnar crystals between layers and tracks but also raise the risk of low-melting grain boundary formation and crack initiation [15]. To tailor the solidified microstructure of LAMed SX superalloy, many researchers have studied the mechanism of columnar-to-equiaxed transition (CET) by mathematical models [16,17,18,19,20] and experimental approaches [21,22,23]. Methods such as adjusting processing parameters [24] and substrate crystallographic orientation [25], induction-assisted heating [26] and inclining the coaxial nozzle [27] are explored to control the CET. Gäumann et al. [19] showed that when layers of CMSX-4 SX superalloy were LAMed on the (100) surface of a SX substrate with the same material, the SGs in each layer can be completely remelted by the adjacent new layer with proper processing parameters, and the multilayer SX material in the repaired zone can achieve an identical crystalline orientation to the base components. They first proved that the platform cracks of worn SX components can be successfully repaired with full SX microstructure by LAM process [28].

Similarly, the tip repair process of the SX turbine blade also needs a multilayer LAM process. Avoiding residual SGs and controlling the dispersion of crystalline orientation in the thin-wall remanufactured tip structure are the crucial points. Vilar et al. [29] deposited a multilayer NiCrAlY material structure on SRR99 superalloy, and confirmed that the multilayer structures deposited in appropriate conditions present a SX nature with small deviation of crystalline orientation. Similarly, Liang et al. [30,31] showed that with proper processing parameters, multilayer thin-wall structure of Rene N5 superalloy with SX microstructure can be LAMed on the SRR99 substrate. Henderson et al. [32] showed that near-net-shape SX tip can be regenerated on worn SX turbine blades with careful choice of LAM process parameters and a suitable substrate-filler material. All these works indicate that the LAM process is capable of repairing or remanufacturing SX components successfully, while the molten pool morphology of LAM process fluctuates congenitally and randomly, which results in the unsteady solidification conditions such as temperature gradient and solidification rate at the solidification interface. Moreover, the heat conduction capacity varies with the incremental height of the LAMed thin-wall structure. All these factors may lead to the diversity and instability of microstructure and associated mechanical properties. To guarantee the mechanical properties of laser repaired or remanufactured SX materials identical to the base SX components, a longstanding challenge is to sustain the monocrystalline nature stably without flaws such as plenty of detrimental precipitations and segregation in the repaired zone. Therefore, synthetically characterizing the microstructure, precipitations, and microsegregation in the laser repaired zone not only contributes to acknowledgment of the laser epitaxial forming technique but also helps us to properly evaluate the LAM process for the repair of damaged SX turbine blade tips, benefiting the laser-based SX-repair or remanufacturing applications.

In this study, a thin-wall structure of SX material was fabricated by LAM process on a SX substrate. The microstructure, especially the diversity of crystal morphology, precipitations and microsegregation in the thin-wall structure were characterized in detail. The effect of a subsequent heat treatment process on the microstructure and precipitations was discussed.

## 2. Materials and Methods 

**Figure 1:**
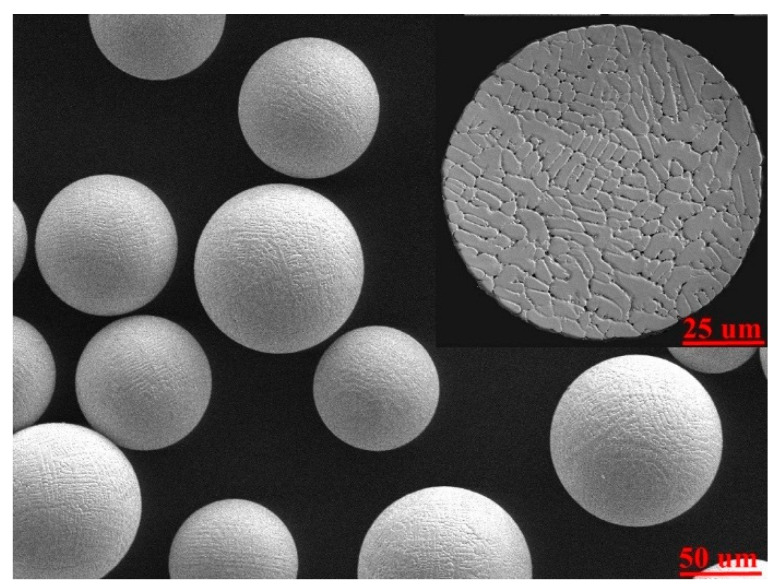
Micrographic for the morphology of SX powder particles, with the insert showing the inner microstructure.

A second-generation nickel-based SX superalloy with a nominal composition of Ni-7.5Cr-9.0Co-1.5Mo-6.0W-6.0Ta-0.4Ti-0.05C-6.5Al-1.0Hf (in wt. %) was used as the material of powders and substrates in the LAM experiments. As shown in Figure 1, the powders manufactured by plasma rotating electrode process (PREP) had a size of 65 μm ~ 110 μm and were mostly spherical in shape. A cast large-volume SX superalloy processed by a standard heat treatment was cut into plate samples with a geometric size of 25 mm × 15 mm × 6 mm in x-axial, y-axial and z-axial directions, respectively. The top surface of substrates is perpendicular to crystalline orientation [001]/<100>, and was ground by sandpaper and cleaned with alcohol before the LAM process.

**Figure 2:**
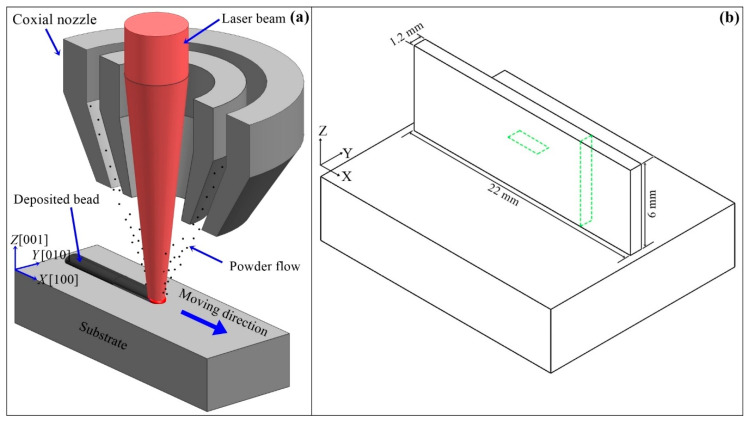
(**a**) The 3D schematic of the LAM process of SX superalloy on the (001) surface of SX substrate and (**b**) the geometrical description of the thin-wall structure.

The 3D schematic of LAM experiment is shown in Figure 2a. The LAM experiment was conducted on the Laser Engineering Net Shaping (LENS) system in the Shenzhen Key Laboratory (China) for Additive Manufacturing of High-Performance Materials. The LENS system is composed of a 3 KW Ytterbium fiber laser, a three-coordinate Computer Numerical Control (CNC) platform, a coaxial nozzle and a double-cylinder powder feeder. The laser beam focused spot diameter at top surface of substrate was about 1.0 mm. The argon gas (purity of 99.99%) was used to shield the molten pool with a flow rate of 10 L/min. The powder particles’ flow was stably transported by argon gas with a flow rate of 6 L/min and focused coaxially with the laser beam. The processing parameters are set as laser power = 350 W, laser moving speed = 10 mm/s, powder feeding rate = 3.5 g/min and incremental height of per layer = 0.2 mm. The ambient temperature of the LAM experiment was about 300 K. The laser beam kept vertical and moved along x-axial direction with unidirectional scanning strategy. When a layer was finished, the laser beam was turned off and the coaxial nozzle moved backward. During the return time, the powder feeding remained stable and the shielding argon gas continuously blew across the bead surface. Finally, tip structures of SX material with a dimension of 22 mm × 1.2 mm × 6 mm in x-axial, y-axial, and z-axial directions respectively were manufactured at the top surface of substrates.

The metallographic samples were cut by wire electrical discharge machining, and then mounted, polished, and etched by a Kalling solution containing CuCl_2_:HCl:C_2_H_5_OH = 5 g:100 mL:100 mL. A standard two-step heat treatment process for traditional cast SX superalloy, which consists of a solution heat treatment with maximum temperature at 1295 °C during 2 h followed by aging with a peak temperature at 850 °C during 24 h, was subsequently performed on the samples. It was carried out in a tubular resistance furnace under a protective atmosphere of argon gas. As shown in Figure 2b, representative observations were conducted in the transverse and vertical sections of the thin-wall structure. The microstructure of metallographic samples was observed by Optical microscopy (OM, Axio Observer 3 m, Carl Zeiss, Germany). The precipitations are analyzed by scanning electron microscopy (SEM, Merlin, Carl Zeiss, Germany). The crystalline orientation of microstructure is studied by electron backscattered diffraction (EBSD, Digiview4, EDAX, Mahwah, NJ, USA). The segregation of element atoms is restructured by three-dimensional atom probe (3DAP, LEAP 4000X HR, CAMECA, Madison, WI, USA). The primary dendritic arm spacing (PDAS) and secondary dendritic arm spacing (SDAS) of the epitaxial columnar crystals, represented by λ_1_ and λ_2_ respectively, were measured on the SEM images observed in the vertical sections (perpendicular to the dominant dendritic stems) of thin-wall structure. The precipitations, as well as the small pores, were recorded by computer aided processing of SEM images observed in the vertical sections and statistically calculated by the MATLAB software (2018a, MathWorks, Natick, MA, USA).

## 3. Results

### 3.1. Microstructure

**Figure 3:**
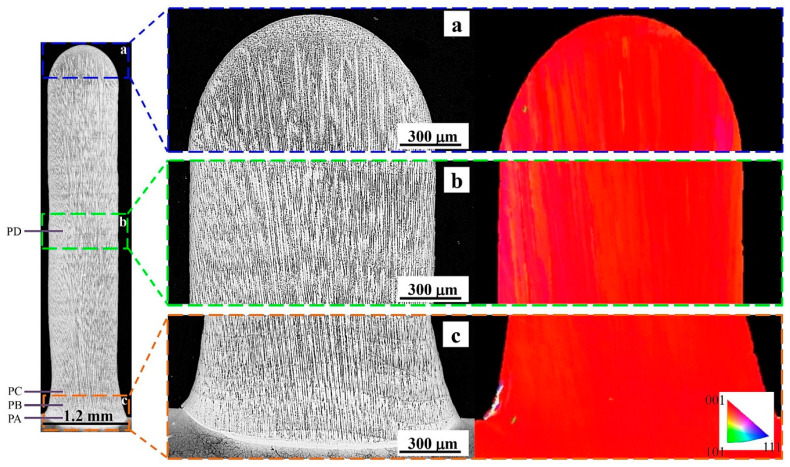
The OM observations and associated EBSD map of microstructure in the transverse section of the thin-wall structure. The magnifications of the (**a**) top; (**b**) middle and (**c**) top regions of OM observations.

**Figure 4:**
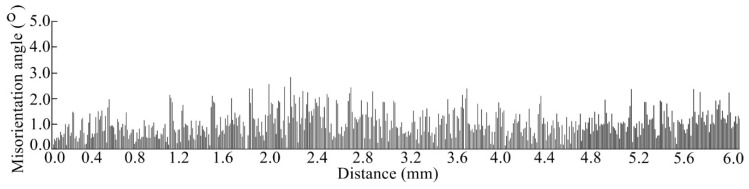
The misorientation angle distribution of microstructure from the bottom to the top of thin-wall structure.

Figure 3 shows the OM observation and associated EBSD map of microstructure in the transverse section of the thin-wall structure. To show more details clearly, three specific regions (bottom, middle, and top) of the OM observed microstructure are selected and amplified. In the OM observations, fine columnar crystals occupy the whole thin-wall structure without distinct SGs. The arrays of long parallel columnar dendrites keep good continuity between plate layers. This is because for the LAM process of SX superalloy with a face-centered cubic lattice, columnar dendrites grows epitaxially with crystalline orientation identical to the substrate. The epitaxial-growth ability of columnar crystals is determined by the supercooling ahead of the crystal tip, and can be evaluated by the |*G*_hkl_|^3.4^/|*V*_hkl_| ratio [24], where *G*_hkl_ is the thermal gradient and *V*_hkl_ is the growth velocity along the crystalline orientation [hkl]/<100>. The thin-wall structure enhances the heat conductive ability along vertical direction and the associated thermal gradient *G*_001_, and simultaneously suppresses the thermal diffusion ability along horizontal direction and the associated *G*_010_ and *G*_100_. Therefore, the columnar dendrites obtain advantageous epitaxial-growth ability along the vertical direction and grow continuously under the given processing parameters. The associated EBSD map shows that the crystallographic orientation of microstructure is identical to the [001]/<100> orientation of substrate, indicating the epitaxial columnar dendrites inherit the monocrystalline nature of substrate. The microstructure coherence is good without any grain boundaries forming between plate layers and substrate. Figure 4 presents the misorientation angle measured from the bottom to top along the central line of the thin-wall structure. The misorientation angle varies in a narrow range of 0° ~ 3°, indicating the SX structure of the substrate is reproduced perfectly in the thin-wall structure. 

**Figure 5:**
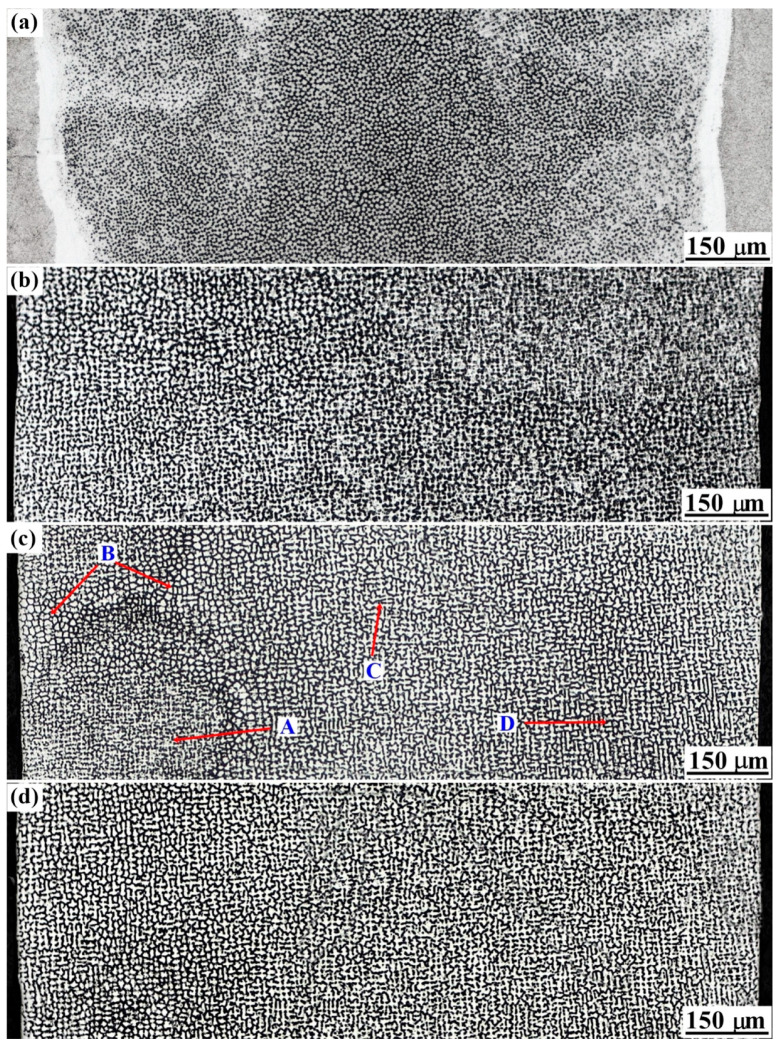
OM observations of crystal morphology in the vertical section of thin-wall structure with a distance of (**a**) 0.0 mm; (**b**) 0.3 mm; (**c**) 0.6 mm and (**d**) 3.0 mm above the substrate surface.

To study the diversity and instability of crystal morphology, the microstructure in the vertical sections was polished, observed and recorded per 0.3 mm from the bottom to the top surface of the thin-wall structure. Four representative sections (PA, PB, PC and PD shown in Figure 3) are exhibited in Figure 5 to analyze the crystal morphology with the manufacturing height in detail. As shown in Figure 5a, in the section PA (0.0 mm above the substrate surface), small columnar crystal stems without any secondary dendritic arms grow perpendicular to the fusion interface of substrate. Due to the curved fusion interface, a featureless zone (white band) at the fusion interface of substrate is also distinct. As shown in Figure 5b, in the section PB (0.3 mm above the substrate surface), the crystal morphology exhibits a diversity. Most of the columnar crystals form distinct secondary dendritic arms, while a minority of columnar crystals only have the primary stems with no or dysplasia secondary dendritic arms. As shown in Figure 5c, in the PC section (0.6 mm above the substrate surface), the diversity of crystal morphology is more obvious than that in the PB plane. Four subregions are marked in Figure 5c to exhibit the diversity clearly. In subregions A and C, the columnar dendrites have well-developed secondary dendritic arms, while the sizes of primary stems and secondary dendritic arms of columnar dendrites in subregion A are smaller than that in subregion C. In subregion B, the columnar crystals mainly have primary stems with no or dysplasia secondary dendritic arms. In subregion D, the columnar dendrites seem to be elongated along the scanning direction. The secondary dendritic arms parallel to the scanning direction are longer than that perpendicular to the scanning direction. Unlike the steady solidification conditions during unidirectional solidification processing of traditional cast SX superalloy, during the LAM process of SX superalloy, the curved solidification interface moves quickly and fluctuates violently, which results in unstable solidification conditions at the crystal tip. The turbulent convection of liquid metal in the molten pool also disturbs the local solidification conditions. All these factors contribute to the diversity and instability of crystal morphology in thin-wall structure. As shown in Figure 5d, the microstructure in the section PD (3.0 mm above the substrate surface) is similar with that in the plane PC. Comparing Figure 5a,d, the diversity and instability of crystal morphology first increases rapidly and then approaches a dynamic balance state with the increase of manufacturing height. 

**Figure 6:**
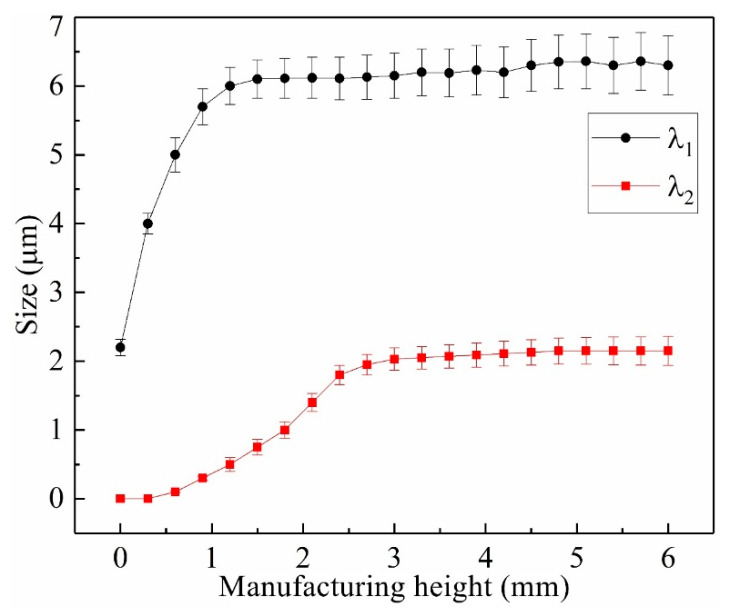
Variation of λ_1_ and λ_2_ of epitaxial columnar dendrites with the increase of manufacturing height of the thin-wall structure.

Figure 6 exhibits the variation tendency of the λ_1_ and λ_2_ of epitaxial columnar dendrites with the increase of manufacturing height. The variation trend of λ_1_ and λ_2_ can be divided into two stages. The average λ_1_ increases quickly from 2.45 μm to 6.15 μm with the increase of manufacturing height from 0 to 1.5 mm, and then fluctuates weakly with the increase of manufacturing height to 6.0 mm. Similarly, the average λ_2_ increases from 0 to 2.05 μm with the increases of manufacturing height from 0 mm to 2.7 mm, and then gradually reaches a quasi-stable state with the manufacturing height exceeding 2.7 mm. The sizes of λ_1_ and λ_2_ of epitaxial columnar dendrites relate to normal thermal gradient *G* and normal solidification velocity *V* at the crystal tip, and can be calculated with the relationships [33]

(1) $$\lambda_{1}=\mu_{1}\times V^{-0.25}\times G^{-0.5}$$

(2) $$\lambda_{2}=\mu_{2}\times V^{-0.3}\times G^{-0.3}$$

where μ_1_ and μ_2_ are material-dependent constant parameters. According to Equations (1) and (2), it is obvious that under the constant solidification velocity *V*, the variation of thermal gradient *G* has a predominant effect on the λ_1_ and λ_2_. During the manufacturing process of the thin-wall structure, the thermal dispersion dominated by large-volume substrate are transitionally governed by the humped thin-wall structure. Accordingly, the heat dispersion capacity around the molten pool decays gradually with the increase of manufacturing height, resulting in the decrease of thermal gradient *G* in the front of solidification interface and resultantly the increase of λ_1_ and λ_2_. When the manufacturing height reaches a certain value, the heat conduction of thin-wall structure achieves a balance, and the thermal gradient *G* and corresponding λ_1_ and λ_2_ reach a quasi-stable state. Therefore, during the LAM of thin-wall structure of SX superalloy, the influence of manufacturing height on the crystal morphology variation shows a local-regional characteristic. According to Figure 6, the effective influence heights of thin-wall structure on the λ_1_ and λ_2_ are 1.5 mm and 2.7 mm respectively with the given processing conditions.

### 3.2. Precipitations

**Figure 7:**
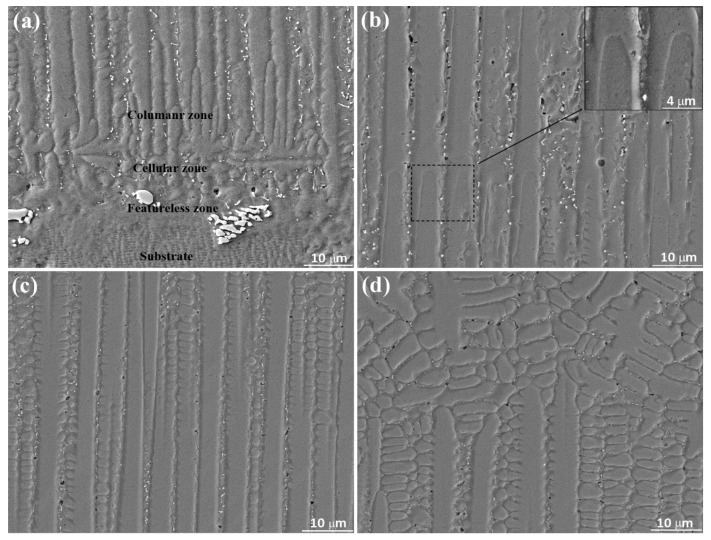
The distribution of precipitations in the transverse section of the thin-wall structure. The SEM images are selected from the (**a**) bottom region; (**b**) layer band region; (**c**) layer inner region and (**d**) top region of thin-wall structure.

**Figure 8:**
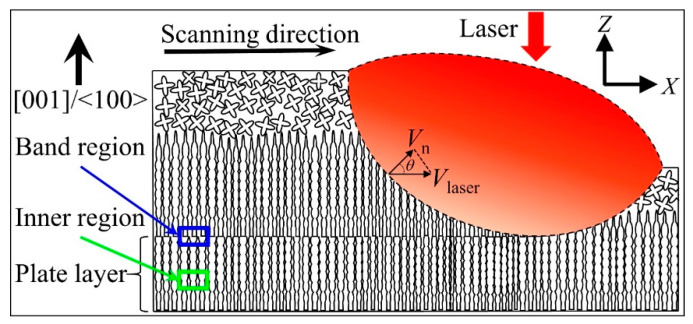
Illustration of the plate layer and geometrical distribution of solidification velocity *V*_n_ at the solidification interface in the representative longitudinal section of the thin-wall structure.

During the layer-by-layer LAM process, the micro inner and band regions of per plate layer, as well as the macro bottom and top of the thin-wall structure, undergo different heat cycle and solidification conditions. The periodic heating-cooling process and the decay of heat dispersion capacity of incremental thin-wall structure diversify the precipitation behaviors including the type, morphology and size in the microstructure. To study the precipitations in the microstructure, the SEM images in the transverse section of the thin-wall structure are shown in Figure 7. As shown in Figure 7a, a featureless zone composed of planner solidification forms at the fusion interface of substrate and is followed orderly by cellular zone and columnar zone. A small number of large and irregular precipitations form in the featureless zone. Plenty of small precipitations mainly form in the interdendritic region of the cellular zone and columnar zone. Figure 7b shows the microstructure in the band region of plate layer. The inserting exhibits the remained outline of partially remelted columnar dendritic tips in the underlying layer. These partially remelted columnar dendrites regrow during the rapid solidification of molten pool in the upper layer, ensuring the perfect continuity and parallelism of SX microstructure between plate layers. A large number of precipitations and discrete pores mainly distribute among the interdendritic regions of columnar dendrites. Figure 7c shows the microstructure in the inner region of plate layer. Only a small number of precipitations and pores form in the interdendritic region of columnar dendrites. The secondary dendritic arms of columnar dendrites are obvious. Comparing Figure 7b,c, it is obvious that the distribution of number and size of precipitations and pores shows periodic band characteristic. Similar to the bamboo, the density of precipitations and pores in the band region are higher than that in the inner region of plate layer. This is because the normal solidification velocity *V*_n_ at the solidification interface geometrically relates to the laser scanning speed *V*_laser_, as shown in Figure 8. The solidification velocity *V*_n_ obtains the value of 0 mm/s at bottom and increases along the solidification interface. In the band region (molten pool bottom) of per plate layer, the low *V*_n_ supports enough time for the atoms rearrangement and the resultant precipitations forming. In the inner region, the increased *V*_n_ leads the mobility of atoms is rapidly reduced and the precipitations do not have enough time to develop. Accordingly, the number and size of precipitations at the band region are greater in number and larger, respectively, than that in the inner region of plate layer. Figure 7d shows the microstructure near the top surface of the thin-wall structure. The epitaxial-growth behavior of columnar crystals is prevented by the SGs. Few of precipitations and pores form in the interdendritic region of epitaxial columnar dendrites and SGs. 

**Figure 9:**
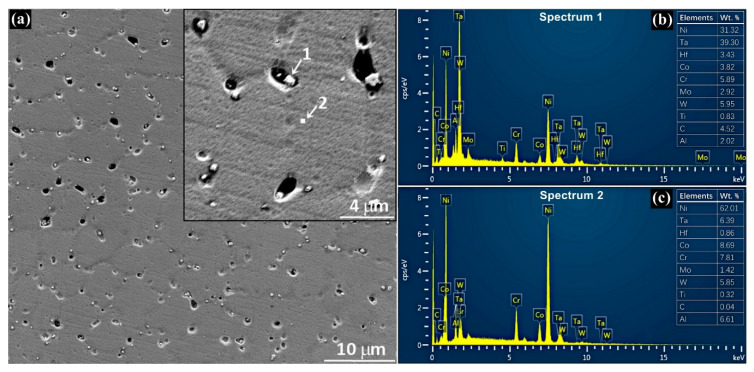
(**a**) SEM micrograph of precipitations and pores near the band region of plate layer in the vertical section of the thin-wall structure, and spectra of (**b**) precipitations (carbides) and (**c**) base matrix.

**Figure 10:**
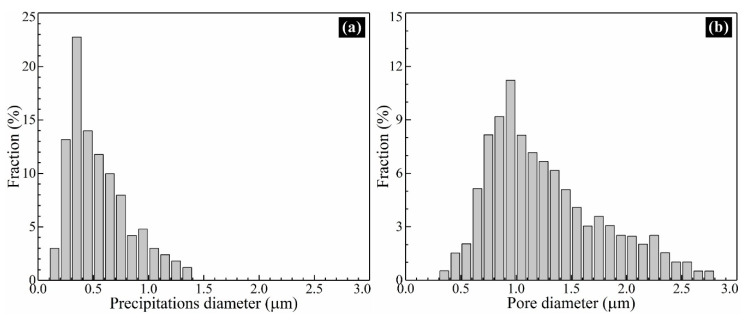
The diameter distribution of (**a**) precipitations and (**b**) pores in the thin-wall structure.

Figure 9 exhibits the distribution of precipitations and pores near the band region of plate layer in the vertical section of the thin-wall structure. The sample was polished and etched slightly in order to distinguish the stems and the interdendritic regions of columnar dendrites. The distribution of precipitations and pores shows symbiosis in the interdendritic region. Almost each pore is accompanied by one or several irregular-shape precipitations. According to the Energy Disperse Spectrometer (EDS) results, the precipitations are of Ta-rich, C-rich and Hf-rich, indicating they are carbides. To analyze the diameter distribution of these precipitations and pores, more than 300 pores and 500 precipitations are recorded and calculated from randomly selected 30 SEM images. The diameter distributions of precipitations and pores are shown in Figure 10. As shown in Figure 10a, the diameter of precipitations distributes in the range of 0.2 μm–1.4 μm and obtains the peak fraction at about 0.4 μm. As shown in Figure 10b, the diameter of pores distributes in the range of 0.3 μm–2.8 μm and obtains the maximum fraction at about 0.9 μm. Comparing Figure 10a,b, it is distinct that the distribution of precipitations and pores shows similar tendency, but the distribution range of precipitations is narrower than that of pores, indicating the size of as-processed precipitations is generally smaller than that of as-processed pores.

### 3.3. Microsegregation

**Figure 11:**
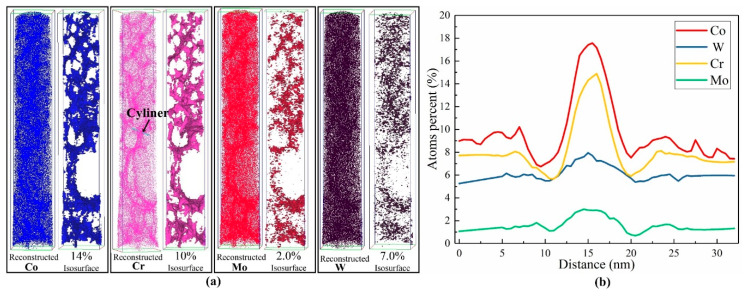
(**a**) 3D distribution of element atoms reconstructed by the 3DAP tomography in the thin-wall structure and (**b**) the concentration variation of representative solute element atoms along the cylinder.

Due to the fast non-equilibrium solidification of laser molten pool, the diffusion time for element atoms is very short. The solute concentration is disturbed by convection of liquid metal and capillarity of mush zone, which may cause the microsegregation in the microstructure. For further understanding the distribution homogeneity of element atoms in the solidified microstructure, a bar sample with a diameter of 70 nm was reconstructed by the 3DAP tomography. Figure 11 shows the 3D visualization of element atoms in the bar sample. The concentration of representative solute element atoms, such as Co, Cr, W, and Mo, is inhomogeneous. According the isosurface with Co > 14%, the concentration of Co element shows a 3D network distribution. The Cr, W and Mo exhibit the similar trend. A cylinder with a length of 35 nm was set to present the detailed variation of elements through the 3D network. The results show that the concentration distributions of Co, W, Cr and Mo keep good consistency, with high value in the 3D network and nearly standard value in the outside region. 

### 3.4. Influence of Heat Treatment

**Figure 12:**
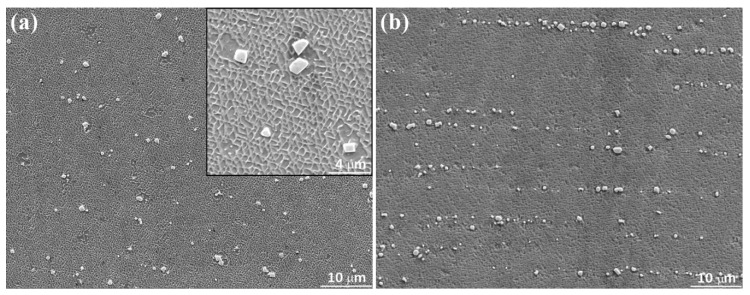
The microstructure after heat treatment in the (**a**) vertical section and (**b**) transverse section of thin-wall structure.

**Figure 13:**
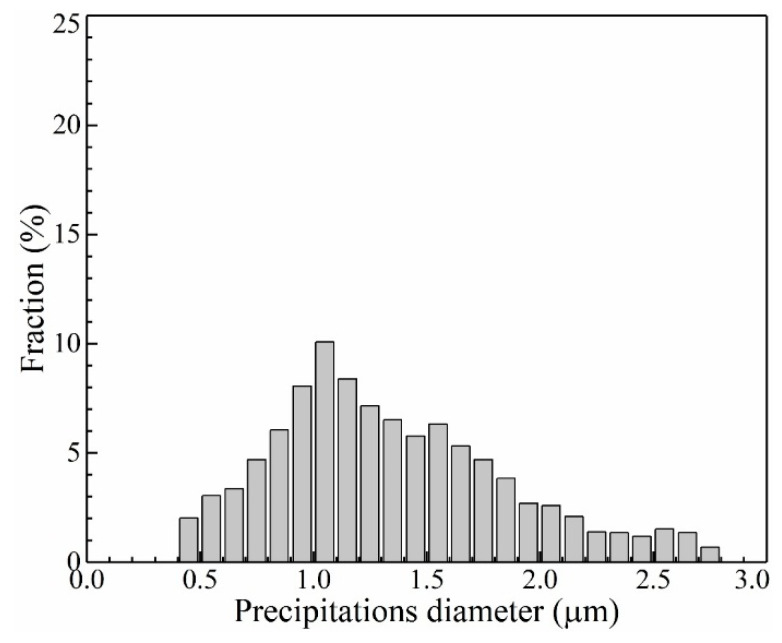
The diameter distribution of precipitations after heat treatment process in the thin-wall structure.

Figure 12 exhibits the SEM images of the microstructure with the subsequent heat treatment. The as-processed pores almost disappears completely. The precipitations have a blocky shape and discretely distribute in the original interdendritic region. The γ’ phases uniformly disperse with a size ranging typically form 0.45 μm–0.9 μm in the γ phase matrix, indicating the heat treatment process for traditional cast SX superalloy is also effective in inducing the precipitation of γ’ in the LAMed SX material. The γ’ phase with coherent cubic morphology of γ matrix is necessary and contributes largely to the high-temperature mechanical properties of SX superalloy, while the γ’ phases in the thin-wall structure present an unwell cubic shape, meaning the heat treatment for traditional cast SX superalloy is not perfectly suitable for the LAMed SX material. Further optimization of the heat treatment process for improving the lattice coherency of precipitated γ’ phase and γ matrix is needed. As shown in Figure 13, the diameter of precipitations after heat treatment distributes in the range of 0.4 μm–2.8 μm and obtains the peak fraction at the 1.0 μm. Comparing Figure 10a and Figure 13, it is distinct that the heat treatment process enlarges the distribution range and size of precipitations, making the diameter with peak fraction increases from 0.4 μm to 1.0 μm.

**Figure 14:**
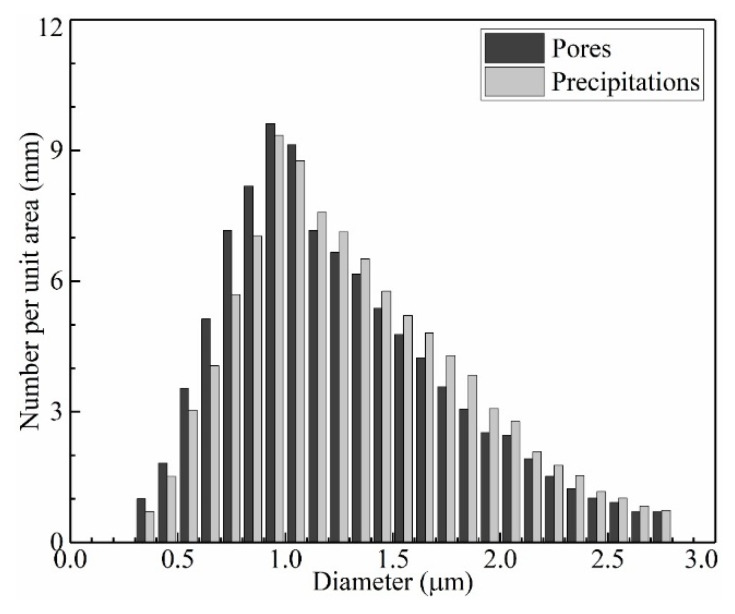
Comparison of diameter distributions between as-processed pores and precipitations after heat treatment in the thin-wall structure.

**Figure 15:**
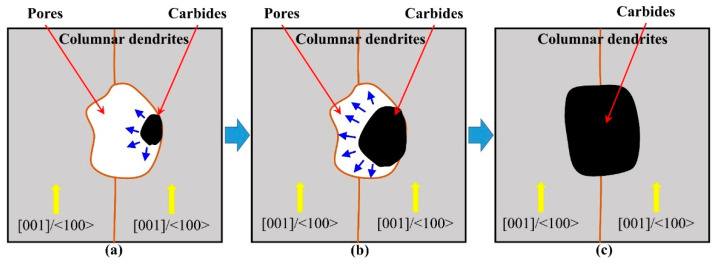
Illustration of the evolution of precipitations and pores in the period of (**a**) initiation; (**b**) rapidly expansion and (**c**) finally occupation during the heat treatment process.

Figure 14 shows the comparison of diameter distributions between as-processed pores and precipitations after heat treatment. It is unique to find that the diameter distribution trends of as-processed pores and precipitations after heat treatment exhibit a reasonable agreement. This is because the heat treatment supports enough time and energy for the atoms’ diffusion. The pores support enough space for the concentration and rearrangement of atoms. The dysplasia as-processed precipitations redevelop during the heat treatment process and finally reach a mature state with a blocky shape. Accordingly, the pores are occupied by the expanded precipitations, as shown in Figure 15.

For the repair of damaged SX turbine blade tips, benefiting the laser-based SX-repair or remanufacturing applications. For the remanufacturing a whole tip of SX turbine blades, the thin-wall structure is practical and it is easy to achieve the monocrystalline nature in the repaired zone. The situation of multi-wall SX structure, especially the morphologic size and associated microstructure formation in molten pool, is more complex. A longstanding challenge is how to sustain the epitaxial-growth continuity of columnar dendrites between tracks and layers stably. Meanwhile, the designing of LAM processing parameters and matching of proper heat treatment parameters should be coupling considered to address the issues of recrystallization of LAMed SX materials in the future. Predictably, the microstructure of multi-wall will also show a diversity.

## 4. Conclusions

In this study, the microstructure, especially the diversity of crystal morphology, precipitations and microsegregation in the LAMed thin-wall structure of nickel-based SX superalloy were characterized and discussed. The influence of a subsequent heat treatment on the microstructure is analyzed. 

Under the given processing conditions, the SX microstructure regenerated perfectly with tolerated misorientation angles in the thin-wall structure. The crystal morphology influenced by the congenital fluctuation of laser additive manufacturing process and the decay of heat dispersion capacity of the incremental thin-wall structure shows obvious diversity and instability. With the increase of manufacturing height, the λ_1_ and λ_2_ of columnar dendrites firstly increase rapidly and then reach a dynamic balance state. The distribution of precipitations and pores keeps symbiosis in the interdendritic region and shows band characteristic with high density in the band region and low density in the inner region of plate layers. The microsegregation of element atoms in the microstructure shows a 3D network distribution. The concentration of element atoms keeps good consistency with high value in the 3D network and nearly standard value in the outside region. The subsequent heat treatment process contributes to the occupation of as-processed pores by the expanded mature blocky shape precipitations. Further optimization of the heat treatment process for improving the lattice coherency of precipitated γ’ phase and γ matrix in the laser additive manufactured SX material is needed and valuable.

## References

[1] Versnyder F.I., Shank M. (1970). The development of columnar grain and single crystal high temperature materials through directional solidification. Mater. Sci. Eng..

[2] Flemings M.C. (1974). Solidification processing. Met. Trans..

[3] Pollock T.M., Tin S. (2006). Nickel-based superalloys for advanced turbine engines: Chemistry, microstructure and properties. J. Propul. Power.

[4] Pollock T., Murphy W. (1996). The breakdown of single-crystal solidification in high refractory nickel-base alloys. Met. Mater. Trans. A.

[5] Babu S., David S., Park J., Vitek J. (2004). Joining of nickel base superalloy single crystals. Sci. Technol. Weld. Join..

[6] Vilar R., Almeida A. (2015). Repair and manufacturing of single crystal Ni-based superalloys components by laser powder deposition—A review. J. Laser Appl..

[7] Saboori A., Gallo D., Biamino S., Fino P., Lombardi M. (2017). An Overview of Additive Manufacturing of Titanium Components by Directed Energy Deposition: Microstructure and Mechanical Properties. Appl. Sci..

[8] Santos E.C., Kida K., Carroll P., Vilar R. (2011). Optimization of laser deposited Ni-based single crystal superalloys microstructure. Adv. Mater. Res..

[9] Rottwinkel B., Pereira A., Alfred I., Noelke C., Wesling V., Kaierle S. (2017). Turbine blade tip single crystalline clad deposition with applied remelting passes for well oriented volume extension. J. Laser Appl..

[10] Kaierle S., Overmeyer L., Alfred I., Rottwinkel B., Hermsdorf J., Wesling V., Weidlich N. (2017). Single-crystal turbine blade tip repair by laser cladding and remelting. CIRP J. Manuf. Sci. Technol..

[11] Vitek J., David S., Boatner L. (1997). Microstructural development in single crystal nickel base superalloy welds. Sci. Technol. Weld. Join..

[12] David S., Vitek J., Babu S., Boatner L., Reed R. (1997). Welding of nickel base superalloy single crystals. Sci. Technol. Weld. Join..

[13] Liu Z., Qi H. (2015). Effects of substrate crystallographic orientations on crystal growth and microstructure formation in laser powder deposition of nickel-based superalloy. Acta Mater..

[14] Gan Z., Yu G., He X., Li S. (2017). Numerical simulation of thermal behavior and multicomponent mass transfer in direct laser deposition of Co-base alloy on steel. Int. J. Heat Mass Tran..

[15] Liu Z., Qi H. (2014). Mathematical Modeling of Crystal Growth and Microstructure Formation in Multi-layer and Multi-track Laser Powder Deposition of Single-crystal Superalloy. Phys. Proced..

[16] Hunt J. (1984). Steady state columnar and equiaxed growth of dendrites and eutectic. Mater. Sci. Eng..

[17] Rappaz M., David S., Vitek J., Boatner L. (1990). Analysis of solidification microstructures in Fe-Ni-Cr single-crystal welds. Met. Trans. A.

[18] Rappaz M., David S., Vitek J., Boatner L. (1989). Development of microstructures in Fe−15Ni−15Cr single crystal electron beam welds. Met. Mater. Trans. A.

[19] Gäumann M., Henry S., Cleton F., Wagniere J.D., Kurz W. (1999). Epitaxial laser metal forming: Analysis of microstructure formation. Mater. Sci. Eng. A.

[20] Liang Y.J., Wang H.M. (2016). Origin of stray-grain formation and epitaxy loss at substrate during laser surface remelting of single-crystal nickel-base superalloys. Mater. Des..

[21] Anderson T., DuPont J., DebRoy T. (2010). Origin of stray grain formation in single-crystal superalloy weld pools from heat transfer and fluid flow modeling. Acta Mater..

[22] Feng L., Huang W., Lin X., Yang H., Li Y., Yang J. (2002). Laser multi-layer cladding experiment on the DD3 single crystal using FGH95 powder: Investigation on the microstructure of single crystal cladding layer. Chin. J. Aeronaut..

[23] Yang S., Huang W., Liu W., Zhong M., Zhou Y. (2002). Development of microstructures in laser surface remelting of DD2 single crystal. Acta Mater..

[24] Liu Z., Qi H. (2015). Effects of processing parameters on crystal growth and microstructure formation in laser powder deposition of single-crystal superalloy. J. Mater. Process. Technol..

[25] Wang L., Wang N., Yao W., Zheng Y. (2015). Effect of substrate orientation on the columnar-to-equiaxed transition in laser surface remelted single crystal superalloys. Acta Mater..

[26] Wang Y., Choi J., Mazumder J. (2016). Laser-Aided Direct Writing of Nickel-Based Single-Crystal Super Alloy (N5). Metal. Mater. Trans. A.

[27] Liu Z., Qi H., Jiang L. (2016). Control of crystal orientation and continuous growth through inclination of coaxial nozzle in laser powder deposition of single-crystal superalloy. J. Mater. Process. Technol..

[28] Gäumann M., Bezencon C., Canalis P., Kurz W. (2001). Single-crystal laser deposition of superalloys: Processing–microstructure maps. Acta Mater..

[29] Vilar R., Santos E., Ferreira P., Franco N., Da Silva R. (2009). Structure of NiCrAlY coatings deposited on single-crystal alloy turbine blade material by laser cladding. Acta Mater..

[30] Liang Y.J., Li J., Li A., Cheng X., Wang S., Wang H.M. (2017). Experimental optimization of laser additive manufacturing process of single-crystal nickel-base superalloys by a statistical experiment design method. J. Alloy. Compd..

[31] Liang Y.J., Cheng X., Li J., Wang H.M. (2017). Microstructural control during laser additive manufacturing of single-crystal nickel-base superalloys: New processing–microstructure maps involving powder feeding. Mater. Des..

[32] Henderson M.B., Arrell D., Larsson R., Heobel M., Marchant G. (2004). Nickel based superalloy welding practices for industrial gas turbine applications. Sci. Technol. Weld. Join..

[33] Stefanescu D.M. (2009). Science and Engineering of Casting Solidification.

